# Thinking ahead – the need for early Advance Care Planning for people on haemodialysis: A qualitative interview study

**DOI:** 10.1177/0269216314560209

**Published:** 2015-05

**Authors:** Katherine Bristowe, Helen L Horsley, Kate Shepherd, Heather Brown, Irene Carey, Beverley Matthews, Donal O’Donoghue, Katie Vinen, Felicity EM Murtagh

**Affiliations:** 1 King’s College London, Cicely Saunders Institute, Department of Palliative Care, Policy & Rehabilitation; 2 Brunel University London, Uxbridge, UK; 3 King’s College Hospital NHS Foundation Trust, London, UK; 4 Guy’s and St Thomas’ Hospital NHS Foundation Trust, London, UK; 5 NHS Improving Quality, Leeds, UK; 6 Salford Royal NHS Foundation Trust, Salford, UK

**Keywords:** End-stage kidney disease, haemodialysis, Advance Care Planning, preferences, priorities, decision making

## Abstract

**Background:**

There is a need to improve end-of-life care for people with end-stage kidney disease, particularly due to the increasingly elderly, frail and co-morbid end-stage kidney disease population. Timely, sensitive and individualised Advance Care Planning discussions are acceptable and beneficial for people with end-stage kidney disease and can help foster realistic hopes and goals.

**Aim:**

To explore the experiences of people with end-stage kidney disease regarding starting haemodialysis, its impact on quality of life and their preferences for future care and to explore the Advance Care Planning needs of this population and the timing of this support.

**Study design:**

Semi-structured qualitative interview study of people receiving haemodialysis. Interviews were analysed using thematic analysis. Recruitment ceased once data saturation was achieved.

**Setting/participants:**

A total of 20 patients at two UK National Health Service hospitals, purposively sampled by age, time on haemodialysis and symptom burden.

**Results:**

Themes emerged around: Looking Back, emotions of commencing haemodialysis; Current Experiences, illness and treatment burdens; and Looking Ahead, facing the realities. Challenges throughout the trajectory included getting information, communicating with staff and the ‘conveyor belt’ culture of haemodialysis units. Participants reported a lack of opportunity to discuss their future, particularly if their health deteriorated, and variable involvement in treatment decisions. However, discussion of these sensitive issues was more acceptable to some than others.

**Conclusion:**

Renal patients have considerable unmet Advance Care Planning needs. There is a need to normalise discussions about preferences and priorities in renal and haemodialysis units earlier in the disease trajectory. However, an individualised approach is essential – one size does not fit all.

**What is already known about the topic?**

- There is a need to improve end-of-life care for people with end-stage kidney disease, including timely and appropriate Advance Care Planning discussions.

**What this paper adds?**

- Haemodialysis patients are rarely given the opportunity to discuss their future care, should their health deteriorate, and describe Advance Care Planning needs before commencing haemodialysis and throughout their disease trajectory.

**Implications for practice, theory or policy**

- There is a need to normalise discussions about preferences, priorities and future care in renal and haemodialysis units, earlier in the disease trajectory.

## Introduction

Over recent years, the end-stage kidney disease (ESKD) population has increased. The incidence of those receiving renal replacement therapy in the United Kingdom has risen from 60 per million population (pmp) (1990) to 108 pmp (2012)^1,2^ and in the United States from 198 pmp (1991) to 348 pmp (2010).^3,4^ The ESKD population is also becoming increasingly elderly, frail and co-morbid,^5^ and the survival of dialysis patients, compared to an age-matched population, is markedly reduced. In the United States, for those aged 50–54 years, survival is 7.1 years on dialysis compared to 27.1 years in the normal population; for those aged 60–64 years, it is 5.1 years (compared to 19.2 years); for those aged 70–74 years, it is 3.6 years (compared to 12.2 years) and for those aged 80–84 years, it is 2.0 years (compared to 6.7 years).^6^

Advance (or Anticipatory) Care Planning (ACP) is a process of discussion between an individual and his or her healthcare provider regarding concerns, goals, preferences, prognosis and future care.^7^ In the UK ACP guidance, renal disease is used as an example where transitions between care phases represent an opportunity to commence ACP. The importance of ACP for renal patients has been highlighted in recent research, particularly regarding symptom burden, quality of life and future care plans.^8^ ACP is most effective when individually tailored, addressing patient and family concerns,^9,10^ and, when appropriately timed, has been found to foster hope among renal patients.^11^ However, current provision of ACP for renal patients is inadequate and inconsistent. Patients report a preference for more information and for ACP to commence earlier in their illness.^9^

Taking into account the changing population, there is a need for a culture shift from a ‘disease-focused’ model towards a ‘holistic care-based’ approach, normalising discussions about preferences, priorities and future care in renal units. The aim of this article is to explore the experiences of haemodialysis (HD) patients regarding starting HD, its impact on quality of life and their preferences for future and end-of-life care, with a view to informing our understanding of the timing and provision of ACP for this population.

## Methods

### Setting

The study setting was two large London renal centres collectively serving approximately 1000 HD patients at 2 main and 10 geographically dispersed satellite units. Both offer a service comparable to other centres nationally, including low clearance clinics (providing advanced kidney care for patients who may need renal replacement therapy within 6–12 months) and <20% of patients presenting late.^12^

### Participants

A total of 20 HD patients were purposely sampled by age (<65, 65 and over), time spent on HD (<12 months, 12–36 months, >36 months) and symptom burden, recorded using a validated symptom measure (Palliative care Outcome Scale–Symptoms (POS-S) renal).^8^ The participants had a mean age of 62 years (median: 62.5 years, range: 25–90 years), mean time spent on HD of 25 months (median: 19.5 months, range: 3–60 months) and mean symptom score of 16 (median: 15, range: 2–35 of a possible 80) (see Table 1). Seven participants attended main HD units and 13 attended satellite units. In all, 11 participants were female and 9 male; 10 were White British, 3 Black African, 4 Black Caribbean and 3 of Asian ethnicity. In the 20 months since completing the study, four participants have died.

**Table 1.** Interview participants.

Participants	20
Age (years)	
<65	11
65 and over	9
Mean	62
Median	62.5
Range	25–90
Gender	
Female	11
Male	9
Ethnicity	
Asian	3
Black African	3
Black Caribbean	4
White British	10
Time spent on haemodialysis (months)	
<12	6
12–36	7
>36	7
Mean	25
Median	19.5
Range	3–60
POS-S renal symptom score	
<10	4
10–20	8
>20	5
Not completed	3
Mean	16
Median	15
Range	2–35
Unit type	
Main	7
Satellite	13
Experience of low clearance clinic	
Yes	16
No	4
Died since participation in study	
Yes	4
No	16

POS-S: Palliative care Outcome Scale–Symptoms

### Interviews

Ethical approval was obtained from the Local Research Ethics Committee (London Riverside NRES – Ref: 11/LO/0286), and all procedures followed were in accordance with Declaration of Helsinki.^13^ Participants were recruited (November 2011–February 2012) through link nurses at each unit who explained the study and introduced the researcher (KB), a sociolinguist with extensive interviewing experience. The researcher further explained the purpose of the study, and each participant gave informed consent before the interview.

The semi-structured interview schedule was guided by a literature review and informed by the multidisciplinary research team and patient and family caregiver advisors. An observational log and field notes for each interview described the following: the flow of the interview, contextual factors, responses from the participant regarding the interview process and questions, and personal reflections. All participants chose to carry out the interview while receiving HD (home or other location also offered), no participants chose to withdraw from the study after consent was taken and many offered to be interviewed again for this or future studies. All interviews were digitally audio-recorded (lasting on average 33 min, range: 13–86 min) and transcribed verbatim, and recruitment continued until data saturation was achieved. Care was taken to use pseudonyms and anonymise any patient, or staff, identifiable references.

### Analysis

Interviews were analysed (by KB and HH) using inductive thematic analysis, which involves five key stages: familiarisation, coding, theme development, defining themes and reporting.^14,15^ Investigator triangulation was used to improve the confirmability of the findings (KB, HH, FM). Emergent themes were reviewed by a person with kidney failure to improve validity. Analysis was managed using N-Vivo qualitative data analysis software (version 10).

## Results

**Figure 1.**
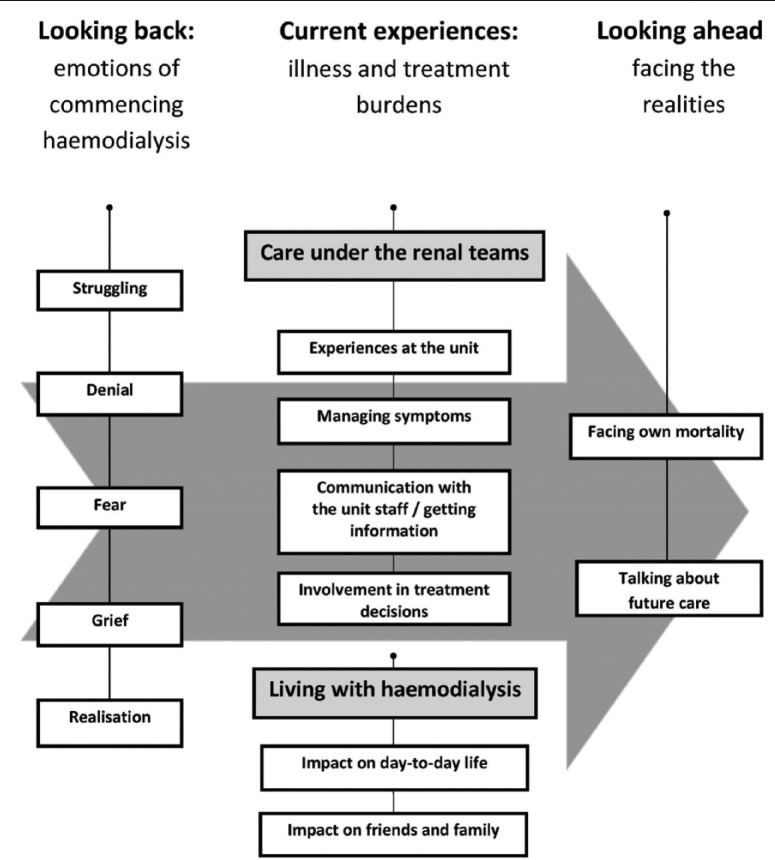
Model of experiences described by people on haemodialysis.

Participants described considerable unmet and unaddressed ACP needs. These needs were broad-ranging; however, specifically they included fear, grief, denial, a shortage of information about their illness and progress, mixed experiences regarding involvement in decisions and a lack of opportunity to discuss their concerns, prognosis and future care. These needs extended from prior to commencing HD and throughout their time on HD. Experiences could be categorised into three temporally discrete main themes (see Figure 1):

*Looking back*: emotions of commencing HD
*Current experiences*: illness and treatment burdens
*Looking ahead*: facing the realities

At all stages, however, the participants described a need for more emotional, psychological and practical support at transitional phases of their disease such as when commencing HD or when deteriorating despite HD.

### Looking back: emotions of commencing HD

Commencing HD was described by all participants, often in highly emotional terms. For many, the experience was associated with fear, sadness and disbelief.

#### Struggling

They described struggling to come to terms with the need to commence this invasive, but seemingly unavoidable, intervention, as recounted by Fiona:

I actually got in touch with the hospice and I was going to go in to palliative care … I just didn’t really want to live anymore because I thought I can’t live a life like this. It was so difficult in the beginning … you wouldn’t imagine how difficult it was. (Fiona, 46, 26 months on HD)

#### Denial

For some participants, there were periods of numbness, disbelief and denial, particularly when first exposed to the HD unit, as explained by Edward:

When I went to the hospital and they were showing me round the unit and they’re showing me the machines and the patients and all I’m seeing is these tubes and the nurse talking to me. And all I’m thinking, I ain’t going on there. That’s all I’m thinking, ah no that’s not me. (Edward, 48, 12 months on HD)

#### Fear

However, there were also periods of intense fear, as described by Carole:

They put a line in me … cause I had to get on the dialysis straight away, then they had the er about doing the bags. Oh, I cried my eyes out, I was terrified when all this at the beginning was going on. I was petrified. (Carole, 55, 47 months on HD)

Fears often had sensory associations: the size and sounds of the machines, the smells of the unit, the sight of the blood in the tubes and the invasiveness of the needles and fistulas.

#### Grief

For many, accepting the life-changing impact of their illness was associated with grief, intense sadness and anger at the loss of their health. For Fiona, this was compounded by an apparent lack of understanding from the HD nurses:

I had a lot of challenges with a lot of nurses at that time and they couldn’t understand why I was crying. They couldn’t understand why I was getting angry. You know I was grieving for my health. (Fiona, 46, 26 months on HD)

#### Realisation

Some participants, however, did describe a degree of acceptance or realisation of the place for HD in their life, albeit often reluctantly. Edward described this point of realisation:

You tend to be in a state of denial … We have to handle ourselves and say, right, we have to do this. There’s going to be days where we don’t want to do it. We’re going to overcome this. We have to really get to realise, this is what’s keeping us alive. (Edward, 48, 12 months on HD)

However, for others, including Carole, acceptance and realisation remained absent due to the unrelenting grief:

I still won’t accept this dialysis. They said I can’t have another transplant. I just can’t accept it. Um, I feel alright yeah, but near enough every day I have a cry at home. (Carole, 55, 47 months on HD)

### Current experiences: illness and treatment burdens

Participants described at length the burden of undergoing HD, the physical environment and care received, the considerable symptom burden, as well as the enormous impact of HD on their life and that of their family.

#### Care under the renal teams

##### Experiences at the unit

Many of the participants described a close and supportive relationship with the nurses and doctors, particularly those, such as Bernard, who had attended for many years:

I’m sitting here eating biscuits a cup of tea and a comfortable chair with a £10,000 machine keeping me alive. The nurses are wonderful, the atmosphere in the place is good. (Bernard, 90, 53 months on HD)

However, for others the experience was marred by the ‘conveyor belt’ culture that pervaded, with prioritisation of ‘getting you on and off’ rather than caring for the individual. This experience was compounded for those reliant on hospital transport, for which there was often a lengthy wait – a 4-h HD session becoming a 12-h ordeal, impacting severely upon recovery the next day.

##### Managing symptoms

Many participants reflected on the busy culture of the unit and the associated lack of opportunity to speak to a doctor. For those who were symptomatic or became unwell while on HD, this was particularly challenging.

##### Communication with the unit staff/getting information

The reported infrequent presence of the doctors also impacted patients’ ability to gain information and explanations about their illness and progress:

I know you’re made aware of what’s happening around you but I think you should be more, more explained to you … I know we get leaflet and things like that, what you should eat and what you shouldn’t eat, but I think you want somebody to really take it in and explain to you in detail. (Victoria, 72, 60 months on HD)

Participants also described a culture of silence when a fellow patient, with whom they had often shared a cubicle for years, no longer attended for HD, as depicted by Bernard:

No they’re very careful of trying not to tell you too much … they would … try and answer a question but without, then suddenly somebody’s not here. (Bernard, 90, 53 months on HD)

##### Involvement in treatment decisions

Experiences of involvement in treatment decisions were very varied. Some, including Victoria, felt they had not been adequately involved, or not in a timely manner:

Could have been more involved. I could have been because I think you’d have to say oh why didn’t they tell me, or why didn’t they tell me there and then I heard at a later date. You want to know here and now. (Victoria, 72, 60 months on HD)

For others, autonomy in treatment decisions was achieved. Rebecca described the decision to return to HD, when her health began to deteriorate after a failed transplant:

It was my decision in the end that I waited and waited and I was far from well by the time I came back. I knew I should come back on but I was just postponing the dreading time I would become a slave to time and machines. No I’ve always been allowed to make my decisions, even when I’d known I’m wrong. (Rebecca, 69, 16 months on HD)

However, not everyone wanted to be involved in treatment decisions. John, for example, actively avoided involvement in decisions, preferring to leave this to the renal care team:

No I just like to, come up here, have this done for 3 hours and I just like to go, get back home … I don’t want to get involved in anything, as long as I’m still breathing and I can get home to my wife that’s all I need. (John, 77, 24 months on HD)

#### Living with HD

##### Impact on day-to-day life

The participants talked openly about the overwhelming impact of kidney failure, and HD, upon their lives and their struggle to accept a new reality. This impact, as described by Carole, was compounded by the cyclical nature of dialysis, with every weekend overshadowed by the foreboding HD routine:

Everything’s changed, every single thing … Well I can’t walk, I can’t eat everything what I fancy, I can’t drink really what I want … to drink. Oh life stinks, horrible, can’t stand it. Terrible times this is. Doesn’t hurt having it done … but oh my god Sunday nights, they’re a git. (Carole, 55, 47 months on HD)

Almost all participants, including Fiona, described the devastating loss of independence and enjoyment in activities:

The dancing, you know I love dancing, I was always on a high. I’ve always loved my music and I’ve always loved dancing, so for me, like apart from reading and visiting places of interest, that seemed to fall away because I was too tired to go anywhere. (Fiona, 46, 26 months on HD)

##### Impact on friends and family

This impact extended to friends and family also, with many participants needing to rely heavily on them:

You know they can’t get on with their life cause I can’t get on with mine, cause I’m stuck on this. Too busy helping me out with my little girl. So it’s a lot of strain and pressure yes on the family and friends. (Denise, 39, 23 months on HD)

However, some participants also described the loss of friends when the illness became apparent and the subsequent isolation.

### Looking ahead: facing the realities

Many participants talked about the future – thinking about their future care and their own mortality.

#### Facing own mortality

They reflected on the death of fellow patients and their fears of becoming unwell while receiving HD, as described by Carole:

Yeah I think about it all the time, you know cause other people have heart attacks you know on the machines. We’ve lost two in this cubicle … But I’m always thinking about it, always. (Carole, 55, 47 months on HD)

However, for Tia, the thoughts regarding her mortality tended to overshadow her time at home, with her family, much to the detriment of her relationship with her husband and daughter:

I have a problem of, I say, before I die. I keep saying that lately, I don’t know why. And it’s really affecting my daughter I need to stop it, but I say before I die I need to do this I need to do that. (Tia, 38, 10 months on HD)

However, some participants did not admit to concerns about mortality, preferring to live for the moment, or not to concern themselves with that which is out of their control. For some participants, such as Margaret, this decision was driven by their faith:

I don’t think about that. I don’t have to think about it, I can’t do nothing about what is going on. So I just, i’m the sort of person that believes in God, so I feel that he that made me knows his purpose. So it’s up to him what he wants to do. I just leave it at that. (Margaret, 59, 12 months on HD)

#### Talking about future care

Many participants described concerns regarding future treatment and hospitalisation, and maintaining attendance for HD, should their health deteriorate. For some, these fears were exacerbated due to underlying concerns about leaving a spouse to cope alone, while for others, including Audrey, the concern was more about the logistics of attending for HD with failing mobility:

Well, the only thing is, what has worried me is, if I couldn’t get out, to come up here I mean. Would they bring me on a stretcher or something like that? I don’t know. Now and again it just wanders through your mind and you think, well we’ll come to that position when we come to it you know. (Audrey, 82, 41 months on HD)

For many of the participants, the lack of opportunity to discuss their concerns about their declining health and future care was compounded by not knowing to whom they should direct their concerns and not wanting to be ‘a bother’. Unless a discussion was instigated by a member of the team caring for them, they would not have an opportunity to raise their concerns.

## Discussion

This study demonstrates the considerable unmet information and ACP needs of people with ESKD throughout their illness. This concurs with pre-existing evidence from Canada and the United States.^9,10,16^ For many participants, the transition to starting HD was abrupt; they felt unprepared for the overwhelming impact of HD, despite most having attended low clearance clinics. This disruption to their life, shattering of hopes and loss of self are described extensively in the chronic disease literature.^17–19^ However, unlike some other disease groups with an unpredictable onset, patients with renal failure usually have the potential to be supported during this period of deterioration to facilitate a stepwise adjustment to life with HD. Indeed, the majority of the patients in this study attended a low clearance clinic. However, instead, the participants described a lack of information or discussion before commencing HD, compounding their shock. This need for earlier engagement in ACP,^11^ and support at transitional phases of illness,^20^ has been described in the literature and could ameliorate emotional, psychological and practical issues associated with the adjustment to life while receiving HD.^21^

Provision of support and discussion of preferences and priorities are particularly important for the younger patients receiving HD. These participants described struggling to maintain a career, family life and roles (spouse, partner, parent or child), alongside HD. For these participants, the ability to maintain these roles was of paramount importance, and they described a need to oscillate between their home self and HD self. This is exaggerated by the fluctuant disease trajectories associated with chronic kidney disease^22^ and the ‘one-day-on, one-day off’ structure of HD. For these patients, the need to commence ACP earlier in the disease trajectory is particularly valuable in order to help them foster realistic hopes and goals.^11^

However, there is also considerable need for ACP among the older patients receiving HD. For those over 65 years, one in four will die within 1 year,^23^ so the need for discussions about preferences and priorities for future care is particularly pressing. In 2005, just under two-third of the UK population reported a longstanding illness, and the population is predicted to continue to age over the next two decades.^24^ It is therefore increasingly important for healthcare providers to understand the complex and evolving needs and preferences of older people with chronic illnesses in order to optimise care and to ensure the most efficient use of services in the future.

The results from this study highlight the importance of ACP and information sharing that is tailored to individual preferences and priorities, as evidenced in previous research.^9^ Although some patients reported a desire to commence discussions about their health, future care and priorities, for some these discussions were not welcome at this stage. Importantly however, many patients receiving HD remain unaware of the supportive care available to them^16^ or even to whom they should direct their concerns. This has been identified in previous research in HD units, describing a focus on ‘nursing the machine’ (attending to the HD process), with little attention to the holistic needs of the patient.^25^ Some possible actions to address these issues could include communication training for HD staff in renal-specific ACP,^26^ regular exploration of patients’ clinical status, symptoms, quality of life, concerns and priorities, perhaps during HD session, to identify those with most need, and annual review with the patient and family to discuss any changes in the last year.^27^

Using qualitative methods, it is not possible to make judgements as to the generalisability of these results. However, purposive sampling was used to capture diversity among participants’ experiences to improve transferability. Investigator triangulation was used to explore the robustness of the analysis, discreteness and interactivity of themes, and to explore deviant cases, to ensure credibility, dependability and confirmability of the findings. Subsequent studies would benefit from a longitudinal approach to explore the evolving nature of preferences and priorities and the shifting role of ACP for this population, as well as the management of transitional phases in renal disease.

## Conclusion

There is a need to normalise discussions about concerns, fears, preferences, priorities and future care for those with kidney failure throughout the renal pathway to enable a culture change to best meet the needs of this population. This can only be achieved by strengthening the support available to those with kidney failure and continued education and training of renal staff to minimise the avoidance of such discussion due to fear of causing distress. Such training should be tailored to highlight the importance of clear information giving, of ACP, where appropriate, and the diverse and evolving needs of this population.
